# Two complementary model-based methods for calculating the risk of international spreading of a novel virus from the outbreak epicentre. The case of COVID-19

**DOI:** 10.1017/S0950268820001223

**Published:** 2020-06-09

**Authors:** Eduardo Massad, Marcos Amaku, Annelies Wilder-Smith, Paulo Cesar Costa dos Santos, Claudio Jose Struchiner, Francisco Antonio Bezerra Coutinho

**Affiliations:** 1 School of Medicine, University of Sao Paulo and LIM01-HCFMUSP, Sao Paulo, Brazil; 2 School of Applied Mathematics, Fundacao Getulio Vargas, Rio de Janeiro, Brazil; 3 School of Veterinary Medicine, University of Sao Paulo, Sao Paulo, Brazil; 4 Department Public Health and Clinical, Heidelberg Institute of Global Health, University of Heidelberg, Heidelberg, Germany; 5 Medicine, Epidemiology and Global Health, Umeå University, SE-901 85 Umeå, Sweden; 6 Department of Disease Control, London School of Hygiene and Tropical Medicine, London, UK; 7 UNIP – Universidade Paulista, Sao Paulo, Brazil

**Keywords:** Coronavirus, mathematical modelling

## Abstract

We present two complementary model-based methods for calculating the risk of international spread of the novel coronavirus SARS-CoV-2 from the outbreak epicentre. One model aims to calculate the number of cases that would be exported from an endemic country to disease-free regions by travellers. The second model calculates the probability that an infected traveller will generate at least one secondary autochthonous case in the visited country. Although this paper focuses on the data from China, our methods can be adapted to calculate the risk of importation and subsequent outbreaks. We found an average *R*_0_ = 5.31 (ranging from 4.08 to 7.91) and a risk of spreading of 0.75 latent individuals per 1000 travellers. In addition, one infective traveller would be able to generate at least one secondary autochthonous case in the visited country with a probability of 23%.

## Introduction

Given the extent of global travel patterns [1–3], newly emerging diseases can rapidly spread globally. In general, respiratory pathogens spread faster [4] than vector-borne viruses [5, 6] or those that require very close contact such as Ebola [7] or Lassa [8]. In late 2019, a novel coronavirus, severe acute respiratory syndrome coronavirus 2 (SARS-CoV-2) emerged in Wuhan, China, which rapidly spread globally [9] and caused epicentres of COVID-19 disease in multiple countries. SARS-CoV-2 has a high reproduction rate and is easily transmitted via respiratory droplets among humans [10]. Population flow data between Wuhan and other major cities in mainland China were clearly correlated with the number of cases exported from Wuhan to other city clusters in mainland China before the lock-down [11]. The potential for rapid international spread via air travel was enormous, and indeed first exportations followed high travel volumes to Thailand, Hong Kong and Singapore [9]. As of 1 June 2020, more than 6 million cases and more than 360 000 deaths due to COVID-19 have been reported in more than 200 countries.

The speed of spread depends on the air passenger volumes, the basic reproduction rate as a measure of transmissibility and the incubation time [12, 13]. In this paper we present two complementary methods for calculating the risk of international spread of a new virus from an epicentre. The first method aims to calculate the number of cases that would be exported from an endemic country to disease-free regions by travellers. The second method calculates the probability that one of the infected travellers will generate at least one secondary autochthonous case in the visited country. The calculation for disease exportation is simpler than the calculation for infection importation. One difference is that in the case of disease importation travellers to endemic areas return infective to their home country, whereas in the case of disease exportation travellers depart from their endemic home country in a latent state. This latter assumption is based on the conjecture that symptomatic individuals do not travel. For asymptomatic travellers, their disease will manifest itself either during the flight or after arrival in the visited disease-free country dependent on the time of infection and incubation time.

In the case of disease importation, the key parameter is the force of infection of the disease in the visited endemic country. In the case of disease exportation, the key parameter is the latency duration of the disease in the travellers' home country. In the case of disease importation latency is not too important and the model considers only susceptible, infected and removed individuals. On the other hand, in the case of disease exportation latency is important because it is assumed that infected and symptomatic individuals are either so sick that they do not manage to travel or are not allowed to board the plane due to exit screening.

## Methods

### The models

#### Model 1. Calculating the number of exported cases from an endemic country

In this section we consider the case of infective travellers from an endemic country visiting a disease-free country, and so exporting the infection to the visited country. Once arriving in the visited disease-free country those infective visitors may trigger an outbreak that can establish itself depending on the value of the basic reproduction number *R*_0_ of the infection in the disease-free country. If *R*_0_ is greater than one, the disease will spread. We will approach the problem with a deterministic formulation.

The model is a classic susceptible-exposed-infected-removed (SEIR) model given by the following set of equations:1
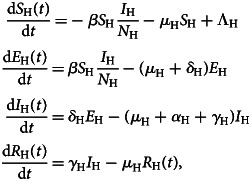
where *S*_H_(*t*) is the number of susceptible individuals, *E*_H_(*t*) is the number of incubating and asymptomatic individuals, who have the disease but do not transmit it, *I*_H_(*t*) is the number of infectious individuals, *R*_H_(*t*) is the number of individuals recovered from infection and *N*_H_(*t*) = *S*_H_(*t*) + *E*_H_(*t*) + *I*_H_(*t*) + *R*_H_(*t*) is the total population. The parameters are *β*, the potentially infective contact rate, *δ*_H_, the inverse of the incubation (or latency) period, *γ*_H_, the duration of infectiousness and *μ*_H_ and *α*_H_ are the natural and disease-induced mortality rates, respectively.

Hence, the number of new infections per unit of time corresponds to the infection incidence, denoted*λ*(*t*) = *βS*_H_(*I*_H_(*t*)/*N*_H_(*t*)).

The basic reproduction number, *R*_0_, that is, the number of secondary infection produced by an infectious individual in an entirely susceptible population along his/her infectiousness period, associated with system (1) is deduced in Appendix A:2
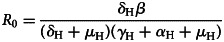


For exportations, our interest is the prevalence of latent infections in the local population, from which, some individuals will travel already infected but not yet symptomatic. We estimated the disease prevalence in the population, that is, the number of infected individuals at each instant of time, *I*_H_(*t*), by integrating the third equation of equation (1) to obtain [3]:3
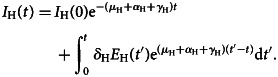


Dividing *I*_H_(*t*) by the size of the local population, *N*_H_, we obtain the prevalence, that is, the proportion of infectious individuals, *p*_I_(*t*), in the endemic country as follows:4
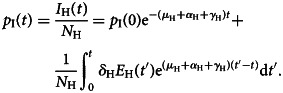


On the other hand, multiplying the number of visitors to a given disease-free country by the prevalence of latent (infected but not infectious individuals), *p*_E_(*t*), generates the number of infected visitors or exportations of infections. Integrating the second equation of (1) yields the following quantity *E*_H_(*t*), exposed or latent individuals:5
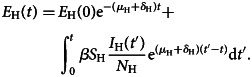


Dividing *E*_H_(*t*) the total population *N*_H_, yields the prevalence of infected but not yet infectious individuals in the home country as follows:6
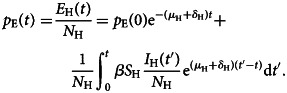


To obtain this prevalence, the force of infection of the disease, that is, the number of new cases of infection per time unit, *β*(*I*_H_(*t*)/*N*_H_), in this endemic region is a necessary input variable. The best information normally available is the notification rate of infectious individuals, *δ*_H_*E*_H_ (this term is the number of individuals that evolve from the latent to the infectious state), provided by disease surveillance systems. Equation (6) will be used later in the paper.

#### Model 2. Calculating the probability of infection introduction in a disease-free country

In this section we calculate the probability that an infected traveller (index case) from an endemic country arriving infective in a disease-free country generates a secondary autochthonous case.

On arriving in the disease-free country each infected visitor will trigger an outbreak that will establish itself depending if the value of the basic reproduction number *R*_0_ of the infection is greater than one. Since we are dealing with a low number of travellers, we need to approach the problem with a stochastic formulation.

This model assumes that a density of one infected individual, *I*_H_(*t*_0_), arrives at *t* = *t*_0_ and remains infective for a period of (*μ*_H_ + *γ*_H_ + *α*_H_)^−1^ days, that is7
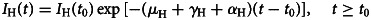
where *μ*_H_, *γ*_H_ and *α*_H_ are the natural mortality rate, the recovery rate from infection and the disease-induced mortality rate, respectively. If the region where these infected travellers arrive had an area *A* the number of them is *I*_H_(*t*_0_)*A*.

The total number of new cases infected by these travellers, Δ weeks after its introduction, New Cases, is given by8
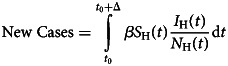
where *β* is the potentially infective contact per unit time between infected and one susceptible individuals, and *S*_H_ and *N*_H_ are the susceptible and total population, respectively.

The risk of New Cases invasion of a previously unaffected country, Risk_new cases_, can be defined as the probability that at least one autochthonous case be produced by the arrival of one single infected individual at the area during his/her infectiousness period. For calculating this risk, we assumed a non-homogeneous simple birth process [5], which describes the propagation of the disease.

Let *P*_*n*_(*t*) be the probability of *n* cases. The probability generating function of such process is 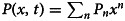. After some calculation, we obtain the probability of *x* cases at time *t* [5, 6]:9
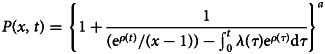
where *a* = *I*_H_(*t*_0_)*A*, *λ*(*t*) = *β*((*I*_H_(*t*_0_)*S*_H_(*t*))/*N*_H_) and 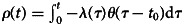.

We have assumed that the region to be studied has an area *A*, the number of infected travellers that arrived at *t* = *t*_0_ is *a* = *I*_H_(*t*_0_)*A*. We set *a* = 1 from now on, that is, a single index case arrives at the non-affected area.

Expanding (9) in powers of *x* we find that the risk, that is, the probability of having *n* infected individuals at time *t*, denoted Risk_new cases_(*n*, *t*) as9*a*
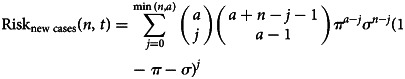


The risk (probability) of having no infected individuals is9*b*
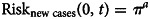


In equations (9a) and (9b) 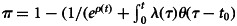 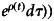 and 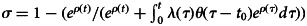.

The probability of at least one autochthonous case in a previously unaffected region can be calculated as the tail probability, that is, the probability of the infection invading the previously non-affected area:10
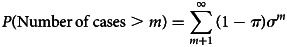
for *m* *=* 1, equation (5) reduces to11
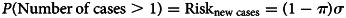


## Results. Illustrating the models

To illustrate the models' performance, we consider the case of the outbreak of COVID-19 in the province of Hubei, China. At the time of writing, this province was responsible for approximately 82% of the total number of COVID-19 cases in the world.

We used data from the WHO website [7]. As we use the case of Hubei outbreak only to illustrate the models, we calculated the incidence of cases in that province by multiplying the total world number of daily cases of infections by 0.84. As China modified the diagnostic criteria along the course of the outbreak, we used incidence data only until 11 February 2020.

### Illustrating model 1

We begin by fitting a continuous function to the daily number of reported cases, that is, the incidence of new cases per time unit, *δ*_H_*E*_H_(*t*). We assume here that new cases are symptomatic cases. This assumption is probably very reasonable for the Hubei epidemic because tests were developed only at the end of the Hubei outbreak. The function has the bell-shaped form:12
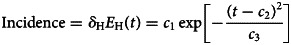
where *c*_*i*_, (*i* = 1, …, 3) are the fitting parameters and *t* is the time.

**Fig. 1.**
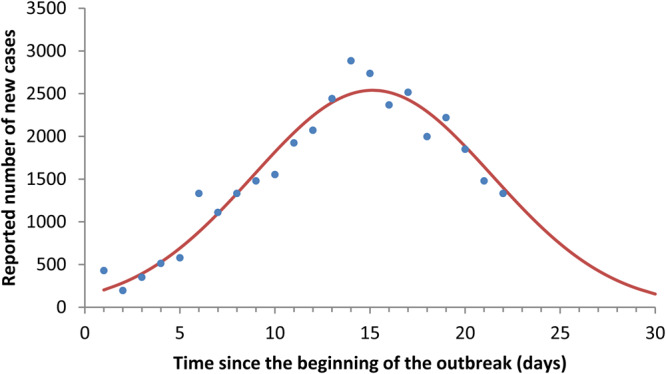
Fitting of reported cases to equation (11).

The fitting of reported new cases per time unit to equation (11) is shown in Figure 1.

Note that equation (12) fits the Hubei COVID-19 cases reasonably well. If equation (12) is inserted into equation (4), the COVID-19 prevalence at each instant of time *I*_H_(*t*) is obtained.

On the other hand, we can fit the initial exponential phase (e^*ϕt*^) of the prevalence curve obtained in Figure 1. It is then possible to estimate the value of *R*_0_ according to (see Appendices A and B):13
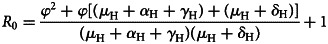
where φ is the rate of the exponential growth and 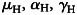 and *δ*_H_ as in equation (1). The parameter values used to calculate *R*_0_ are shown in Table 1.

**Table 1.** Parameter's values to calculate *R*_0_ as in equation (13)

Parameter	Value	Comments
*ϕ*	0.232	Fitted to the Hubei data (see Fig. 2)
*μ*_H_	3.91 × 10^−5^ per day	Life expectancy of 70 years
*α*_H_	7.73 × 10^−4^ per day	Lethality of 3.2%
*γ*_H_	0.2 per day	Duration of symptoms of 5 days
*δ*_H_	0.167–1.0 per day	Latency ranging from 1 to 6 days

**Fig. 2.**
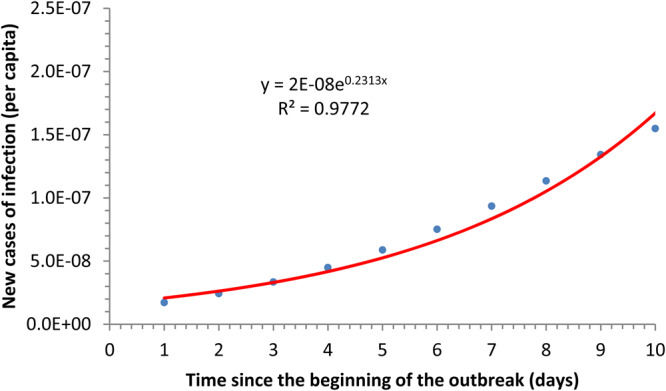
Fitting the new cases per capita to an exponential function, the data from Hubei province.

The value of the estimated *R*_0_ resulted in an average of 5.31 (ranging from 4.08 to 7.91) for the outbreak in the province of Hubei. Figure 2 shows the fitting of data from Hubei province to an exponential function.

It is possible, in principle, to fit the parameters of system (1) in order to retrieve the prevalence curve. The parameters then can be used to estimate the number and the prevalence of latent individuals (equations (5) and (6)). Alternatively, taken the COVID-19 average latency period of 3 days (i.e. *δ*_H_ = (1/3) per day), *E*_H_(*t*) can be calculated by simply dividing equation (11) by *δ*_H_, that is,14
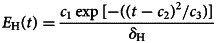
from which it is possible to estimate the prevalence of asymptomatic latents in the population:15
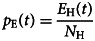


**Fig. 3.**
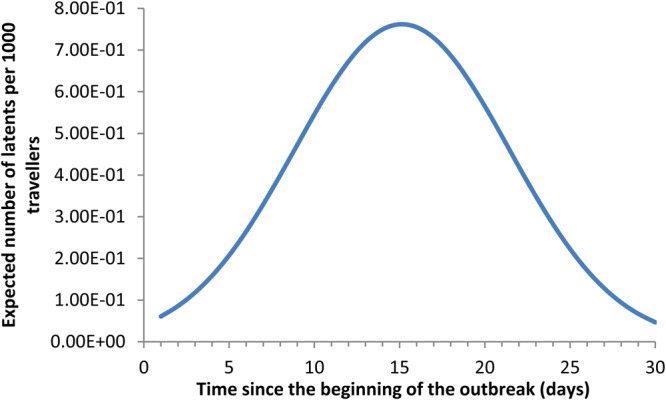
Expected number of latent travellers.

The result for the case of COVID-19 in the province of Hubei is shown in Figure 3.

As an example in a cohort of travellers that depart from Hubei at week 15 the relative number of latent individuals carrying the COVID-19 virus is of 0.75 individuals per 1000 travellers, which is much higher than Ebola, for instance [7]. In other words, out of 1333 travellers from that region, 1 would be infected.

### Illustrating model 2

Next, we assume that one individual traveller from an endemic country (index case) visits a disease-free country and remains infective for a period of (*μ*_H_ + *γ*_H_ + *α*_H_)^−1^ days.

To calculate the probability of a secondary autochthonous case generated by each infected traveller, we used the incidence curve described above to calculate the probability generating function according to equation (9) where the incidence is represented by the parameter *λ*(*t*).

Next, we calculate the values of parameters *π* and *σ* from equations (9a) and (9b) to estimate the probability that the infected traveller who imported the virus to his/her home country would generate at least one secondary case, according to equation (11). The result is 23%, that is, one single infective traveller would be able to generate at least one secondary case along his/her infectiousness period, with probability of 23%. Note that the expected number of secondary case is the average value of the basic reproductive rate, that is, 5.31.

## Discussion

In this paper we propose two complementary models for calculating the risk of international spreading of the novel coronavirus SARS-CoV-2 from the initial epicentre of COVID-19 in Wuhan, China. One model addresses the case of disease exportation from the epidemic outbreak and considers a certain number of travellers leaving the epidemic region during the incubation period, thereby importing the virus into another country. The model is deterministic and was illustrated with the data from the initial outbreak in the province of Hubei in China.

The first model's simulation resulted in an average *R*_0_ = 5.31 (ranging from 4.08 to 7.91) and a risk of spreading of 0.75 latent individuals per 1000 travellers. If we consider the monthly number of travellers from the city of Wuhan described by Wu *et al*. [14] to other Asian countries of around 86 000, we should expect almost 65 cases of the infection to these countries.

The second model addresses the case of the probability of disease introduction in a disease-free country by an index case from the epidemic epicentre. The model considers the situation in which a single infected traveller from an epidemic region, acquires the infection and travels to a disease-free country where he/she can trigger a local outbreak. As we consider a single traveller we approach the case with a stochastic formulation. We simulated the model with the same case of the province of Hubei in China and the results show that one single infective traveller would be able to generate at least one secondary autochthonous case in the visited country, along his/her infectiousness period, with a probability of 23%. This probability should be contrasted with the average number of secondary cases the traveller would generate at his/her home country of 5.31. The latter is the average basic reproduction number of COVID-19 in the community of Hubei and should not be essentially different elsewhere when the population is immunologically naïve and there is a homogenously mixing pattern of contact. In a stochastic context, even when *R*_0_ is greater than 1, there is a probability of extinction of the infection. Moreover, the 23% risk of exportation means the probability that one traveller when arriving in the infectious condition would generate at least one secondary autochthonous case of COVID-19.

Some important limitations are worthwhile mentioning about our approach:

Our model assumes that only latent individuals travel. However, it is possible that some mildly symptomatic cases can escape from the screening measures at the moment of the travel. Moreover, for example, a number of the earliest known exported cases travelled when sick. There are reports of travellers taking antipyretics to mask their fever, and then board the plane. However, the number of patients who travel with mild symptoms is likely to be very small when compared to the non-symptomatic latent individuals and this should not interfere with our results. Furthermore, our results will depend on the incidence of COVID-19 in the departing country. For example, during the peak of the COVID-19 outbreak in Europe, about 3−6% of air passengers were SARS-CoV-2 positive on repatriation flights [15].

From the modelling perspective an important limitation is the homogeneously mixing assumption. We are well aware of the many heterogeneities involved in transmission of a directly transmitted pathogen like SARS-CoV-2. In addition, the deterministic approach of the exportation model is an approximation of the real dynamics involved in transmission. However, both limitations above do not invalidate the qualitative results of the models. Considering the large number of people involved in the current epidemic the deterministic approach and the homogeneously mixing assumption can be considered as a good first approximation of the problem. However, heterogeneities can be introduced in the model using the techniques described in [16], and these heterogeneities could have significant influences in the quantitative results of our model. For instance, if variation in infectiousness would be included, the risk of spread could be lower on average, and the speed of the infection spread could be affected as well.

Equation (1) assumes that only infected individuals, *I*_H_, are infectious. In fact, at least a fraction *f* of the exposed individuals, *E*_H_, may be infectious. So *I*_H_ in the first and the second equations of system (1) should be replaced with *I*_H_ + *fE*_H_. Equation (A6) in Appendix A shows that *e*_H_(0) and *i*_H_(0) are related. This artificial feature can be removed by adding to equations (A1) and (A2) the initial infection terms 
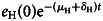
 and 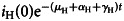, respectively, and solving them [10].

Another limitation of our approach concerns the data upon which we exemplify our application. We have access only to the global number of cases from the WHO website on COVID-19 and in order to apply the model for the province of Hubei we assumed that that region represents 84% of the global cases. Hence, we assumed a direct proportionality of the cases to simulate the model on these data. In addition, we simulate the model until 11 February because China modified the diagnostic criteria along the course of the outbreak. The incidence curve, however, had already started to wane at that time. Moreover, we used the incidence data for the province of Hubei only to exemplify the models, which could be applied, in principle, to other situations related to the spread of pathogens from outbreak epicentres.

Finally, it should be commented that the model assumes that the potentially infective contact rate *β* in the receptive country of the index cases is the same as in the province of Hubei. In fact, *β* has a remarkable seasonality, being higher during winter time, declining throughout spring time into summer time. Therefore, the probability of autochthonous cases is super-estimated for receptive countries during the summer season.

We believe that the models presented here may present a significant step forward in estimating the risk of importation of the novel coronavirus SARS-CoV-2.

## Appendix A

**Finding the basic reproduction number given by equation (2)**

Linearising the second and the third equations of the system (1) around the no-disease state, that is, *S*_H_ = *N*_H_ − *S*_H_, *E*_H_ = 0 + *e*_H_, *I*_H_ = 0 + *i*_H_ and *R*_H_ = 0 + *r*_H_ when *S*_H_, *e*_H_, *i*_H_ and *r*_H_ are small, we obtain

A1

A2

Assuming

A3

A4

we get

A5

A6

The above system has non-trivial solution if the determinant of the unknown *c*_1_ and *c*_2_ are different from zero. We then obtain

A7

Equation (A5) has a positive solution when

A8

is greater than 1. Thus, *R*_0_ is the basic reproduction number.

Also,

A9

## Appendix B

Now we relate the basic reproduction number (equation (A5)) to the rate *φ* of new cases *N*_c_(*t*) given by equation

B1

The rate *φ* is obtained by fitting equation (B1) to the initial exponential growth shown in Figure 2.

Using equation (A4) we have that the number of new infected cases at *t* = 0 is given by

B2

Since *e*_H_(*t*) and *i*_H_(*t*) are given by equations (A3) and (A4) and *λ* is the positive root of equation (A7), we have

B3

and

B4

But

B5

from equation (A2). So,

B6

## References

[1] Glaesser D (2017) Global travel patterns: an overview. Journal of Travel Medicine 24(4). doi: 10.1093/jtm/tax007. 28637267

[2] Tuite AR (2020) Global trends in air travel: implications for connectivity and resilience to infectious disease threats. Journal of Travel Medicine, taaa070. doi: 10.1093/jtm/taaa070. 32374834

[3] Kraemer MUG (2020) Mapping global variation in human mobility. Nature Human Behavior. 10.1038/s41562-020-0875-0 32424257

[4] Fineberg HV (2014) Pandemic preparedness and response − lessons from the H1N1 influenza of 2009. New England Journal of Medicine 370, 1335–1342. 24693893 10.1056/NEJMra1208802

[5] Redondo-Bravo L (2019) Imported dengue in Spain: a nationwide analysis with predictive time series analyses. Journal of Travel Medicine 26(8), taz072. doi: 10.1093/jtm/taz072. 31608405 PMC6927315

[6] Halstead S and Wilder-Smith A (2019) Severe dengue in travellers: pathogenesis, risk and clinical management. Journal of Travel Medicine 26. 10.1093/jtm/taz062 31423536

[7] Tuite AR (2019) Ebola virus outbreak in North Kivu and Ituri provinces, Democratic Republic of Congo, and the potential for further transmission through commercial air travel. Journal of Travel Medicine 26. 10.1093/jtm/taz063 31414699

[8] Wolf T (2020) Fifty years of imported Lassa fever − a systematic review of primary and secondary cases. Journal of Travel Medicine, taaa035. doi: 10.1093/jtm/taaa035. 32219400

[9] Bogoch II (2020) Potential for global spread of a novel coronavirus from China. Journal of Travel Medicine 27. 10.1093/jtm/taaa011 PMC7074660 31985790

[10] Liu Y (2020) The reproductive number of COVID-19 is higher compared to SARS coronavirus. Journal of Travel Medicine 27. 10.1093/jtm/taaa021 PMC7074654 32052846

[11] Lau H (2020) The positive impact of lockdown in Wuhan on containing the COVID-19 outbreak in China. Journal of Travel Medicine 27(3), taaa037. doi: 10.1093/jtm/taaa037. 32181488 PMC7184469

[12] Massad E (2018) Estimating the probability of dengue virus introduction and secondary autochthonous cases in Europe. Scientific Reports 8, 4629. 29545610 10.1038/s41598-018-22590-5 PMC5854675

[13] Lopez LF (2016) Modeling importations and exportations of infectious diseases via travelers. Bulletin of Mathematical Biology 78, 185–209. 26763222 10.1007/s11538-015-0135-z PMC7089300

[14] Wu JT, Leung K and Leung GM (2020) Nowcasting and forecasting the potential domestic and international spread of the 2019-nCoV outbreak originating in Wuhan, China: a modelling study. Lancet (London, England). doi: 10.1016/S0140-6736(20)30260-9. PMC7159271 32014114

[15] Lytras T (2020) High prevalence of SARS-CoV-2 infection in repatriation flights to Greece from three European countries. Journal of Travel Medicine 27. 10.1093/jtm/taaa054 PMC7184451 32297940

[16] Coutinho FAB (1999) Modelling heterogeneities in individual frailties in epidemic models. Mathematical and Computer Modeling 30, 97–115.

